# Only time will tell – why temporal information is essential for our neuroscientific understanding of semantics

**DOI:** 10.3758/s13423-015-0873-9

**Published:** 2016-06-13

**Authors:** Olaf Hauk

**Affiliations:** MRC Cognition and Brain Sciences Unit, 15 Chaucer Road, Cambridge, CB2 7EF UK

**Keywords:** Semantics, Neuroimaging, Time course, Embodied cognition

## Abstract

Theoretical developments about the nature of semantic representations and processes should be accompanied by a discussion of how these theories can be validated on the basis of empirical data. Here, I elaborate on the link between theory and empirical research, highlighting the need for temporal information in order to distinguish fundamental aspects of semantics. The generic point that fast cognitive processes demand fast measurement techniques has been made many times before, although arguably more often in the psychophysiological community than in the metabolic neuroimaging community. Many reviews on the neuroscience of semantics mostly or even exclusively focus on metabolic neuroimaging data. Following an analysis of semantics in terms of the representations and processes involved, I argue that fundamental theoretical debates about the neuroscience of semantics can only be concluded on the basis of data with sufficient temporal resolution. Any “semantic effect” may result from a conflation of long-term memory representations, retrieval and working memory processes, mental imagery, and episodic memory. This poses challenges for all neuroimaging modalities, but especially for those with low temporal resolution. It also throws doubt on the usefulness of contrasts between meaningful and meaningless stimuli, which may differ on a number of semantic and non-semantic dimensions. I will discuss the consequences of this analysis for research on the role of convergence zones or hubs and distributed modal brain networks, top-down modulation of task and context as well as interactivity between levels of the processing hierarchy, for example in the framework of predictive coding.

## Motivation

The question of how our minds represent and process information about the external world, such as objects, actions, people, and events, in order to make inferences from observations, predict future events, or communicate with other individuals, is central to most areas of cognitive science (Barsalou, 2008; Harnad, 1990; Searle, 1980). Cognitive neuroscience addresses this issue by attempting to reveal the neural code that underlies semantic representations and processes. For empirical research to be successful, theoretical developments about the nature of semantic representations and processes should go hand-in-hand with a discussion of how these theories can be tested and distinguished on the basis of empirical data – we should ask questions that we can answer. Most of the major debates, for example about the role of distributed brain systems in semantics, or about the existence and localization of convergence zones and hub regions, or about the influence of top-down control on semantic processing, are still waiting to be concluded. A detailed review of the empirical literature on these issues is beyond the scope of the present paper, and I do not attempt to draw any of these debates to a conclusion here. Instead, I attempt to make a simple and general but important point, namely that the temporal resolution of our measurement modalities can affect our ability to resolve these theoretical debates.

Usually, functional imaging results are not interpreted in isolation. It is widely acknowledged that multimodal evidence is required. For example, functional and anatomic neuroimaging have been used to confirm and refine neuropsychological findings, for example to explain why damage to certain brain systems disrupts certain cognitive functions (Binder & Desai, 2011; Patterson, Nestor, & Rogers, 2007; Price, 2000). The most straightforward and fortunate case of “converging evidence” is when different methods indeed provide the same result. However, it is more likely that different methods produce at least slightly, if not substantially, different outcomes, and the researcher is faced with the question of what method to trust for what kind of conclusion.

Most current neuroscientific research on semantics is based on metabolic neuroimaging, especially functional magnetic resonance imaging (fMRI; see, e.g., reviews: Binder, Desai, Graves, & Conant, 2009; Jobard, Crivello, & Tzourio-Mazoyer, 2003; Taylor, Rastle, & Davis, 2013; Visser, Jefferies, & Lambon Ralph, 2010). A major assumption behind this research is that localization can tell us something about function (Henson, 2005; Page, 2006). For example, in the area of grounded cognition, activation in sensorimotor systems is supposed to indicate that semantic representations are grounded in sensorimotor representations (Barsalou, 2008; Pulvermuller, 2013). However, there are a number of reasons why different methods may be sensitive to very different physiological phenomena, or on different spatial and temporal scales. Even a seemingly simple concept such as “brain activation” can be highly complex – does it refer to blood oxygenation, blood volume, firing rates, synchrony, or the amplitude of oscillations in the gamma band, the beta band, etc. (Singh, 2012)? From this it follows straightaway that the search for the best method is an ill-posed problem – the question should state “best for what purpose?” The methodology should be chosen on the basis of the theory being tested, not vice versa – we should first decide what kind of information we need to address our research questions, and then check out the options. Unfortunately, there is still a huge explanatory gap between our theories about higher-level cognition and the neural mechanisms that implement them (Carandini, 2012; Embick & Poeppel, 2014).

Here, I would like to elaborate on a point that – in its generic form – has been made many times before: if the brain processes we are interested in are fast, then our measurements should be fast. This point is probably articulated more often by electroencephalography (EEG), magnetoencephalography (MEG), and electrocorticography (ECoG) researchers than in the fMRI, PET, or NIRS communities. In the following, I emphasize where temporal information is essential for conclusions about functions and computations in semantic processing (but not restricted to semantic processing). The conclusion will not be that all questions require temporal information, or that those that do would not benefit from other types of information. However, a satisfactory conclusion to some current issues in cognitive neuroscience does require temporal information about brain activation. These issues involve the detection of semantic hubs, distributed networks, distinguishing top-down from bottom-up processing, and distinguishing feed-forward from feedback processing.

## Spending or wasting time

### Generic arguments

A generic argument in favor of spatio-temporal methods compared to metabolic imaging is that perceptual and cognitive processes occur very rapidly. Behavioral data already demonstrate that visual lexical and semantic categorization processes, from the retina to button press execution, must be completed within half a second or faster (Amsel, Urbach, & Kutas, 2013; Hauk, Coutout, Holden, & Chen, 2012; Ratcliff, Gomez, & McKoon, 2004). Topographies in spontaneous or task-related EEG/MEG can change within tens of milliseconds (Lehmann, Pascual-Marqui, & Michel, 2009).

**Fig. 1:**
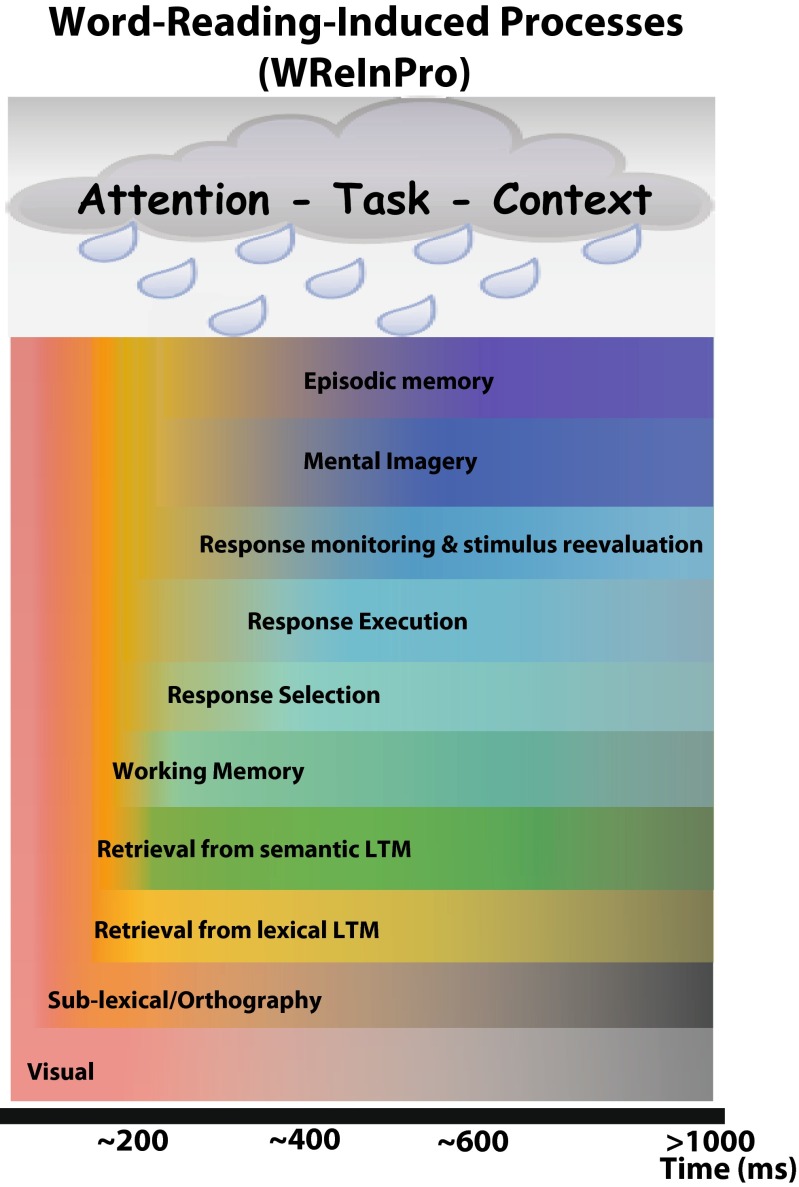
Schematic diagram of Word-Reading-Induced Processes (“WReInPro”). The WReInPro diagram lists processes and computations likely evoked by monomorphemic single-word reading during the first second after stimulus onset. A possible time-course is illustrated based on studies discussed in the main text, but a detailed empirical review of the literature is not within the scope of this paper. *LTM* long-term memory

Any model attempting to explain behavioral or brain data should take into account everything that may systematically affect the dependent variable. In the case of fMRI, where brain activation is integrated across several seconds due to the slow hemodynamic response function (Buckner, 1998), the model therefore has to account for processes of up to several seconds, i.e., well beyond the button press in most perceptual and cognitive tasks. For EEG and MEG, the model needs to account for processes up to the latency chosen by the researcher, which may even be before stimulus presentation or before or after a behavioral response. Considering that response latencies in common lexical and semantic decision tasks as well as in overt naming are often below 1 s, this raises the question of how to account for the remaining seconds in fMRI data. Furthermore, EEG and MEG studies have shown that psycholinguistic variables, such as word length or frequency, can affect the brain response at multiple latencies (e.g. Amsel, 2011; Hauk, Davis, Ford, Pulvermuller, & Marslen-Wilson, 2006). It is currently not clear whether these effects occur in the same or different brain regions, and it is possible that they at least overlap. Preempting the following sections, Fig. 1 provides an illustration of word-evoked computations and processes likely to occur within the first second after stimulus onset.

Temporal resolution would be less of a limitation for fMRI if the brain was strictly feed-forward, and in a given processing sequence every brain region activated only once at a particular stage. On the basis of independent knowledge, one might be able to label different regions as “early visual cortex” or “frontal executive control areas.” However, recurrent activation can affect early visual areas at later stages (Ahissar & Hochstein, 2004; Lamme & Roelfsema, 2000; Woodhead, et al., 2014), and even simple stimuli rapidly activate large brain networks, including frontal cortex and limbic structures (Bar, et al., 2006; Bullier, 2001; Morris, et al., 1998).

Irrespective of the duration of perceptual and cognitive processes, it has been argued that brain networks communicate at very short temporal scales, represented by different frequency bands up to the gamma band (around 30–40 Hz) or high gamma (100 Hz and beyond) (Canolty, et al., 2006; Engel & Fries, 2010; Siegel, Donner, & Engel, 2012). If this is the case, then separating different networks in different frequency ranges, and establishing phase relationships among areas with those networks, requires imaging modalities with millisecond temporal resolution. While non-spectral connectivity measures exist (Valdes-Sosa, Roebroeck, Daunizeau, & Friston, 2011), this requirement will obviously be the same for any method that relies on information at these temporal scales.

### What counts as a “semantic” effect?

It is difficult to disagree with the previous point that if millisecond temporal resolution is required, then electrophysiological recordings are the methods of choice. But is millisecond temporal resolution essential in order to study semantics? The fundamental question is whether an observed effect that is assumed to be the result of the manipulation of a semantic stimulus or task variable can really be interpreted as a “semantic effect,” and if so what aspect of semantics does it represent. In this section, I discuss different views on representations and processes that affect the interpretation of neuroimaging results as semantic. I do not argue that there is a unique decomposition of semantics into particular processes and representations, or that any of the elements have to be clearly separable from each other in terms of brain regions or systems. But whatever this decomposition is for a particular study, one needs to address the question of whether effects of non-semantic aspects of task or stimuli may be misinterpreted as semantic. For simplicity, I focus my examples on lexical semantics, but the same issues are relevant for semantics at the phrase or sentence level.

The distinction between representation and process has a long tradition in cognitive science. Marr (1982) defined a mental representation as "a formal system for making explicit certain entities or types of information, together with a specification of how the system does this," i.e., for a representation to be meaningful one needs to know the processes that may operate on it. Anderson (1978) argued that every dependent variable will reflect properties of stimulus representations and the processes that operate on them, and that these two may therefore be impossible to distinguish.

The term “(semantic) representation” is used ubiquitously in the neuroscientific literature on semantics – but what is a semantic representation? If we ask about how meaning is represented in semantic memory, then this refers to our acquired knowledge about facts and events stored in declarative long-term memory. Where do we store the information that a rose can sting, a stein is for drinking beer, and that marmots are funny little creatures when we are not thinking or talking about them? Unfortunately, we cannot see these long-term representations directly. In any given experiment, we can only measure the result of processes that operate on these representations for specific stimuli, tasks, and contexts. Based on a single experiment, whether resulting in response time distributions or brain activation patterns, it is impossible to conclude with certainty that the observed effects reflect the inherent structure of long-term representations. This “access versus storage” problem was already a topic in the neuropsychologic literature before the time of neuroimaging (Rapp & Caramazza, 1993; Warrington & Shallice, 1979). It is true for all measurement modalities that their data will reflect an amalgamation of long-term memory representations and the processes that act upon them in the specific task context.

Relevant task-related information from long-term memory is accessed through some form of retrieval process, which to a certain degree will involve executive control processes (Thompson-Schill, D'Esposito, Aguirre, & Farah, 1997; Whitney, Kirk, O'Sullivan, Lambon Ralph, & Jefferies, 2012), and may depend both on stimulus type and task. For example, familiar and unfamiliar stimuli or meaningful and meaningless stimuli may rely on executive control processes to different degrees (Hoffman, Jefferies, & Ralph, 2011). Retrieving different kinds of information from the same stimuli may involve different brain regions (Lee, et al., 2002; Rogers, Hocking, Mechelli, Patterson, & Price, 2005). Do pseudowords engage the same retrieval processes such as words, or do they activate the corresponding brain systems more strongly because one has to “try harder” to find a meaning (Mechelli, Gorno-Tempini, & Price, 2003)? Or not at all because they do not have a meaning? A difficult decision (e.g., whether or not to accept a low-frequency word as a word or to reject a very word-like pseudoword) may trigger additional types of processes, for example, spell-checking as suggested for visual word recognition (Balota & Chumbley, 1984). As long as these processes depend on the prior classification of the stimulus, timing will help – decision-related processes should occur later in the brain response. For example, early effects may rule out explanations in terms of spell-checking or stimulus re-evaluation.

Semantic information from long-term memory is usually retrieved for a purpose. In the context of a specific task, task-relevant information needs to be held in working memory in a format that allows optimal decision making. In natural reading, information needs to be available in order to be integrated into context, or it might be discarded if irrelevant. Working memory has a structure, and for example differs between verbal and pictorial stimuli (Baddeley, 2003), but potentially also between word categories (Shebani & Pulvermuller, 2013). It is possible that distributed brain systems representing associations of words or objects with individual sensorimotor experiences support working memory processes. This may be a basis for claims of some authors that early activation of sensorimotor systems during word processing does not prove their involvement in semantic processing, but reflects a parallel process that provides a “coloring” of the concept (Mahon & Caramazza, 2008). In this case, distinguishing early from later effects will not solve the problem either, since working memory representations may be required early-on in processing.

Stimulus processing does not stop with the behavioral response or completion of the task. We usually do not read words or look at objects just in order to complete a 2AFC task, but to think about the meaning, its relevance to the current situation, to integrate it into a wider context etc. The corresponding processes may involve “mental imagery” (Paivio, 1986). Several neuroimaging studies have made attempts to demonstrate that what they found is “semantics” rather than “imagery” (Gold, et al., 2006; Hauk, Davis, Kherif, & Pulvermuller, 2008; Wheatley, Weisberg, Beauchamp, & Martin, 2005; Willems, Toni, Hagoort, & Casasanto, 2010). The imagery argument has been an important motivation for EEG/MEG studies on semantics, since the earlier a semantic effect occurs, the less likely it is to reflect mental imagery (Hauk, Shtyrov, & Pulvermuller, 2008). Drawing the line between semantics and imagery is a contentious issue in itself, since imagery may be required for mental simulations involved in higher-level conceptual processing (Barsalou, 2008). Here, my point is that it is important to be clear about where on the continuum between these two interpretations one operates. This has implications for neuroimaging research: Word and pseudoword differences in fMRI data that some interpret as semantic may actually reflect “thoughts evoked by a word,” and differences between animals and tools may be due to imagining a playful dog compared to thinking about Do-It-Yourself work. The involvement of sensorimotor cortex in imagery processes, for example, motor cortex for motor imagery (Jeannerod & Frak, 1999) and visual cortex for visual imagery (Kosslyn, 2005), is well established. Such imagery effects may vary systematically with semantic variables of interest, and may for example be stronger for concrete or highly imageable words, and differ in content between living and non-living things, actions and objects, etc.

In a similar vein, words may evoke the retrieval of episodic memories. When presented with the word “hammer” or “dog” without any context, one may start imagining how one once hit one’s thumb with a hammer, or when one last played with a dog. The type of episodic memory as well as its strength are likely to differ between meaningful and meaningless stimuli, but may also be different among semantic categories. Pseudowords may not evoke any episodic memories at all, while objects and tools may evoke episodic memories and associated imagery that produce differences in brain activation in different sensorimotor areas. As for imagery, these processes are likely to occur late in processing, and may be ruled out with appropriate temporal information.

The computations and processes discussed above are summarized in the “WReInPro” (Word-Reading-Induced Processes) diagram in Fig. 1. This diagram also suggests a possible time course for these processes based on studies discussed in this paper, but a detailed review of the empirical literature must be left to future publications. The main purpose of this diagram is to illustrate that any behavioral or brain response at a certain latency reflects a number of sequential or parallel processes that may be confounded with semantic variables.

### Who is on top of whom? Interactivity, top-down and bottom-up processing

In the context of reading, some authors have claimed that “one of the oldest debates in visual word recognition concerns the demarcation between bottom-up and top-down processing” (Carreiras, Armstrong, Perea, & Frost, 2014). When we say “To someone with a hammer, everything looks like a nail,” we seem to suggest that our goal or context shapes our perception, i.e., stimulus processes at the earliest stages. This question is not specific to language. The question of whether top-down effects manifest themselves as “filtering” of the input or “selection” after the perception process has already been asked decades ago (Deutsch & Deutsch, 1963; Treisman & Riley, 1969).

The term top-down modulation has been used in at least two different ways in the literature. It may refer to the effects of non-stimulus-related variables such as task or context on stimulus processing, for example, when word processing is compared between a semantic and non-semantic task (e.g., Chen, Davis, Pulvermuller, & Hauk, 2013, 2015). It may also refer to the interactivity of brain systems at different levels of the processing hierarchy. Brain systems at a higher level of the processing hierarchy may modulate those at a lower level during stimulus processing, for example, when comparing connectivity of frontal and inferior temporal areas between words and pseudowords (e.g., Woodhead, et al., 2014). I will discuss the “task modulation” and “interactivity” interpretations separately.

Some authors treat semantics as inherently task-dependent, because the relevant perceptual simulations need to be situated in a specific context (Barsalou, 2008). Others describe semantics as an automatic activation of networks that have evolved on the basis of associative learning (Pulvermüller, 1999). These two views may be different sides of the same coin: Barsalou’s theory explicitly distinguishes an early linguistic stage from a later simulation stage. The former may well be task independent, and correspond to Pulvermüller’s automatic ignition of cell assemblies. Also, these cell assemblies may receive excitatory and inhibitory modulation depending on task requirements, and task-relevant cell assemblies may remain active for a longer period (Pulvermuller, 2013).

In the behavioral literature on visual word recognition, the task modulation of psycholinguistic effects such as the word frequency effect has been taken as evidence for flexible, rather than automatic, word processing (Balota & Yap, 2006). fMRI studies have shown that activation patterns to the same words or pictures change with respect to task demands (Lee, et al., 2002; Rogers, et al., 2005; van Dam, Rueschemeyer, Lindemann, & Bekkering 2010). These data indicate that different decisions are based on different types of information, and that stimulus processing differs between tasks. Following the arguments presented in the previous sections, this does unfortunately not answer the crucial question as to whether task demands affect early perceptual processing or later decision making, or an interaction of the two. Recent EEG/MEG studies have demonstrated that task demands can affect early brain responses (Chen, et al., 2013, 2015; Strijkers, Bertrand, & Grainger, 2015). Importantly, they may affect brain responses differently in different latency ranges: Chen et al. (2015) reported task modulation of imageability and word frequency effects before 250 ms, but task-independent effects at later latencies, predominantly in the anterior temporal lobe. Averaging activation across all latency ranges would not be able to reveal whether task-dependent or task-independent effects come first. Moreover, short-lived early effects might be missed.

Top-down modulation may also refer to interactivity of “low-level” regions by “high-level” regions, for example, the modulation of visual areas by inferior frontal cortex, or of posterior temporal regions by anterior temporal lobe. This requires the interaction of brain regions at a fast temporal scale. It has been proposed that interactions or connectivity among brain regions are achieved via oscillations in different frequency bands (Siegel, et al., 2012). For example, the beta band (approx. 15–25 Hz) has been associated with top-down control in visual attention paradigms, and gamma band responses (above 30 Hz) with bottom-up processing (Engel & Fries, 2010). Predictive coding has been presented as a general framework for neural stimulus processing, as a continuous computation of prediction errors between the predictions of higher levels of the hierarchy with evidence from lower levels (Bastos, et al., 2012). Irrespective of the details of these theories, if gamma and beta band activity reflect qualitatively different processes in different brain networks as fundamental as top-down and bottom-up processing, then separating these fundamentally different processes from each other requires temporal resolution in the millisecond range. Furthermore, if the connectivity among brain regions is (at least partly) reflected in phase relationships between oscillations, which reflect temporal delays on the millisecond scale, then this is the temporal resolution required during measurement. This is also the case if connectivity is not reflected in spectral measures – all that matters is that these mechanisms operate on very short time scales.

## Conclusion

How is the above relevant to current debates on the neuroscience of semantics? Here, I briefly summarize and elaborate on the examples already used in the previous sections.

To what degree does semantic processing rely on amodal convergence zones or hubs, and to what degree on distributed sensorimotor systems? The original proposal of convergence zones included the possibility of multiple convergence zones in different brain areas (Damasio, 1989; Meyer & Damasio, 2009). Semantic dementia research in combination with neuroimaging has led to the proposal that anterior temporal lobes (ATLs) serve as unique semantic hubs (Patterson, et al., 2007; Rogers, et al., 2004). Other authors have suggested a special role of angular gyrus (AG) for semantic processing (Binder & Desai, 2011). An important feature of a hub region should be that it activates early on in processing, either before or at least at the same time as other brain areas contributing to semantics, such as sensorimotor cortex. Consistent early activation of one particular region, such as ATL or AG, would justify the hub interpretation compared to multiple convergence zones. It may also be possible that there are different hubs at different times, for example, an early hub for the fast retrieval of lexical semantics, and later hubs for context-dependent simulations, combinatorial semantics, etc.

It is another important feature of a hub region that it binds together distributed brain regions, for example, sensorimotor areas in the hub-and-spoke model (Patterson, et al., 2007; Rogers, et al., 2004). The role of sensorimotor areas in semantics is still a contentious issue in the literature on embodied cognition (Barsalou, 2008; Hauk & Tschentscher, 2013; Mahon, 2014; Pulvermuller, 2013). In order to test whether hubs and distributed brain regions activate simultaneously and are functionally and effectively connected, temporal information is essential. In the fMRI literature, the interpretation of semantic category differences is plagued by the “imagery” confound (Hauk, Davis, et al., 2008). It may be difficult to delineate at what point mental simulations contributing to semantics end and deliberate mental imagery begins. However, if sensorimotor activation were to be observed at the time of a button press in a semantic categorization task, one would have to argue that this activation did not contribute to the execution of this task. Temporally distinguishing different levels of semantics should be part of future theoretical developments.

It is currently not clear how to best capture connectivity in semantic brain networks, for example whether it is reflected in “oscillations” using spectral connectivity measures such as coherence or phase-locking values. In any case, if the question is whether changes in activity in one brain area cause near-simultaneous activity changes in another brain area, then there is no way around temporal resolution. This is equally important to study interactions between brain regions associated with different levels of the processing hierarchy, such as top-down effects from frontal to visual regions (Woodhead, et al., 2014). If predictive coding proves to be a productive framework for semantic processing, the precise predictions of this theory about continuous computation of prediction errors between different levels of the processing hierarchy, and the possible role of oscillations in different frequency bands, can only be addressed with methods of sufficient temporal resolution.

Similarly, revealing the mechanisms of top-down modulation by task and context requires the temporal resolution to distinguish early perceptual from later decision-related processes, and the possible interaction between the two. The same brain area may activate at several processing stages, and, for example, early perceptual areas can be modulated late in processing by recurrent activation (Lamme & Roelfsema, 2000). Distinguishing early top-down modulation from late recurrent activation required temporal information in the range of tens of milliseconds.

The above analysis of temporal aspects of semantics also has consequences for stimulus selection and experimental design. Finding “semantic” brain regions by contrasting meaningful with meaningless stimuli may produce meaningless results, especially for metabolic imaging, considering the various confounds from word form frequency to mental imagery and episodic memory. However, this contrast has been widely used in the past, as documented in several fMRI meta-analyses (Binder, et al., 2009; Jobard, et al., 2003; Taylor, et al., 2013). Some of these issues can be avoided or minimized by focusing on semantic word categories, or using multiple continuous predictor variables together with possible confounds in parametric designs (Chen, et al., 2015; Hauk, Davis, et al., 2008). Even in this case, confounding factors such as mental imagery and episodic memory need to be addressed, which is best achieved by focusing on brain activity in the appropriate latency ranges.

One criticism that affects spatial and spatio-temporal neuroimaging methods alike is that they are correlational. Whether a brain area activates early or late still cannot tell us whether it supports a particular process, or is just an epiphenomenon. Nevertheless, temporal information can at least narrow down the possibilities. Connectivity measures provide more detailed information about brain networks that can be tested with computationally inspired neuroanatomic models of semantics. However, the ultimate test for causality is to disturb a brain region and test the effect on behavior. In healthy participants, this can be accomplished using transcranial magnetic stimulation (TMS; Devlin & Watkins, 2007). TMS offers the possibility to stimulate specific brain regions at specific time points. For the stimulation to be sensitive, these regions and latencies have to be chosen very carefully. Temporal information about the target processes is essential for the definition of sensitive stimulation latencies.

The obvious methods with high temporal resolution are EEG, MEG, ECoG, and intracranial recordings. These methods themselves have important differences, for example, with respect to sensitivity and spatial resolution that cannot be discussed in detail here. Some of the issues I discussed in the preceding sections require a combination of spatial and temporal information, for example, in order to separate hubs from spokes. Others only require temporal information, for example, to separate early from late top-down modulation, but would benefit from additional spatial information, for example, to localize these effects along the ventral stream. Here, I focused on the temporal domain since it has so far received less attention in the literature than spatial localization. One possible reason for this imbalance, and for the absence of reviews or meta-analyses of the type provided for fMRI studies, is the relatively less standardized analysis and presentation of EEG and MEG results, especially when combined with source estimation (but see Gross, et al., 2013; Picton, et al., 2000, for guidelines). For the neuroscience of semantics, I hope that a more detailed analysis of semantics with respect to temporal aspects, in combination with the development of standardized analysis tools for sophisticated spatio-temporal analysis of brain activity, will bring us closer to answering the questions that motivated this paper – only time will tell.
